# Supplementary material for: Ultrasensitive Detection of Bacillus anthracis by Real-Time PCR Targeting a Polymorphism in Multi-Copy 16S rRNA Genes and Their Transcripts
Source: Int J Mol Sci. 2021 Nov 12;22(22):12224. doi: 10.3390/ijms222212224 (PMC8618755; doi:10.3390/ijms222212224)
Supplement: Supplementary file 1 [file ijms-22-12224-s001.zip › ijms-1441924-SI.pdf]

Ultrasensitive detection of *Bacillus anthracis* by real time PCR targeting a polymorphism in multi-copy 16S rRNA genes and their transcripts

Peter Braun[1], Martin Duy-Thanh Nguyen[1], Mathias C. Walter[1] and Gregor Grass[1]*

[1]Bundeswehr Institute of Microbiology (IMB), Munich, Germany; martin2nguyen@bundeswehr.org (MDTN); peter3braun@bundeswehr.org (P.B); mathias1walter@bundeswehr.org (MCW); gregorgrass@bundeswehr.org (G.G.)

* Correspondence: gregorgrass@bundeswehr.org; Tel.: +49-992692-3981

6. Supplementary Material

6.1. Supplementary Tables

Table S1: Temperature optimum.

Annealing temperature (°C)	Ct-values (Ø±SD)*	Fluorescence (arbitrary units)**
61	33.1±0.1	27.9±0.9
62	32.7±0.2	32.1±1.1
63	32.5±0.2	28.0±0.9
64	32.7±0.1	25.2±0.4

* averages (Ø) ± standard deviations (SD) of n = 3 tests; ** value at the end of the 42th PCR cycle.

Table S2: Primer titration.

Primer concentrations (µM)	Ct-values (Ø±SD)*	Fluorescence (arbitrary units)**
0.5 /0.5	26.6±0.9	13.8±0.4
0.25 / 0.5	26.5±0.5	9.7±0.4
0.5 / 0.25	27.2±0.1	11.7±0.2
0.25 / 0.25	26.6±0.1	6.0±0.4
1 / 0.5	27.6±0.2	12.8±0.6
0.5 / 1	27.3±0.2	15.0±0.4
1 / 1	26.6±0.7	13.0±0.5

* Average (Ø) ± standard deviations (SD) of n = 3 tests; ** Value at the end of the 42th PCR cycle.

Table S3: Probe titration.

Probe concentration BA* / BC** (µM)	Ct-values (Ø±SD)***	Fluorescence (arbitrary units)****
0.25 / 0.25	27.5±0.3	15.9±0.2
0.125 / 0.25	27.5±0.2	9.2±0.5
0.25 / 0.125	27.8±0.1	16.2±0.1
0.125 / 0.125	27.4±0.6	9.9±0.5
0.5 / 0.25	27.3±0.6	24.8±1.6
0.25 / 0.5	27.5±0.3	14.7±0.3
0.5 / 0.5	27.7±0.4	23.2±1.7

* 6-FAM-labeled (hybridizes against the *B. anthracis*-specific allele of the 16S rRNA gene); ** „dark" probe lacking fluorescent label (hybridizes against the *B. cereus* allele of the 16S rRNA gene); *** averages (Ø) ± standard deviations (SD) of n = 3 tests; **** value at the end of the 42th PCR cycle.

Table S4: Magnesium ion titration.

$MgCl_2$ concentration (mM)	Ct-values (Ø±SD)*	Fluorescence (arbitrary units)**
1	28.8±0.3	2.8±0.3
2	27.5±0.7	10.0±0.5
3	28.1±0.4	23.3±1.9
4	27.8±0.4	21.3±1.2

* averages (Ø) ± standard deviations (SD) of n = 3 tests; ** value at the end of the 42th PCR cycle.

Table S5: Pipetting error (5%).

Reaction volume (µl)	Ct-values (Ø±SD)*	Fluorescence (arbitrary units)**
19	28.6±0.4	47.3±6.0
20	28.9±0.1	46.4±3.8
21	29.1±0.1	44.5±5.4

* averages (Ø) ± standard deviations (SD) of n = 3 tests; ** value at the end of the 42th PCR cycle.

Table S6: Intra assay variability*.

Strain	Assay 1	Assay 2	Assay 3	Ct-values (Ø±SD)**	CV***
L3-2641	32.4	32.7	33.1	32.7±0.3	1.0
L3-2649	34.3	33.8	33.6	33.9±0.3	1.1
L3-2657	33.8	33.9	33.9	33.9±0.1	0.2
L3-2665	31.7	31.9	31.9	31.8±0.1	0.4
L3-2866	31.7	31.9	31.9	31.8±0.1	0.4
L3-3391	24.2	24.2	24.3	24.2±0.0	0.3
L3-3399	22.8	22.7	22.7	22.7±0.0	0.1
L3-3407	23.4	23.4	23.3	23.4±0.0	0.2
L3-3408	22.9	22.9	22.9	22.9±0.0	0.3
L3-3415	22.4	22.4	22.4	22.4±0.0	0.0
L3-3416	23.5	23.6	23.7	23.6±0.1	0.3
L3-3421	22.5	22.4	22.2	22.4±0.2	0.2

* replicates (n=3) of each PCR reaction with target DNA were run on the same day; ** averages (Ø) ± standard deviations (SD) of n = 3 tests; *** coefficient of variation (%).

Table S7: Inter assay variability*.

Template copies	Assays day 1	Assays day 2	Assays day 3	Ct-value (Ø±SD)**	CV***
10^4	25.9; 25.8; 26.0	26.2; 26.3; 26.3	25.6; 25.6; 25.6	25.9±0.3	1.2
10^2	32.3; 32.9; 32.3	32.9; 33.0; 32.8	32.4; 32.2; 32.0	32.5±0.3	1.1

* replicates (n=3) of each PCR reaction with target DNA were run on three consecutive days; ** averages (Ø) ± standard deviations (SD) of n = 3 x 3 tests; *** coefficient of variation (%).

Table S8: Probe titration in the presence of 10^9 copies of 16S-BC-allele (absence of 16S-BA-allele).

Probe concentration BA*/BC** (µM) [BA/BC]	Ct-values	Fluorescence (arbitrary units)****
0.25 / 0.25 [1:1]	-***	1.6±0.0
0.25 / 0.5 [1:2]	-***	1.1±0.4
0.25 / 0.75 [1:3]	-***	0.82±0.4
0.25 / 1.0 [1:4]	-***	1.4±0.3

* 6-FAM-labeled (hybridizes against the *B. anthracis*-specific 16S-BA-allele of the 16S rRNA gene); ** „dark“ probe lacking fluorescent label (hybridizes against the *B. cereus* 16S-BC-allele of the 16S rRNA gene); *** no regular amplification, no CT-value; **** value at the end of the 42th PCR cycle.

Table S9: Optimized probe concentration for the detection of the 16S-BA-allele (100 target copies) in the presence of increasing copies of the alternative BC-allele.

Assay Number	16S-BC-allele concentration (copies per reaction)*	Ct value (Ø±SD)**	Fluorescence (arbitrary units)***
1	10^8	****	3.63±0.3
2	10^7	****	3.44±0.2
3	10^6	****	3.35±0.1
4	10^5	33.4±0.6	3.8±0.1
5	7.5×10^4	34.0±1.3	4.8±0.7
6	5×10^4	35.0±0.4	5.2±0.5
7	2.5×10^4	34.2±0.3	6.9±0.3
8	10^4	33.4±0.8	8.6±0.8
9	10^3	32.6±0.1	17.9±0.6
10	10^2	32.9±0.1	25.7±0.7
11	no BA. only 10^5 BC	****	2.6±0.1
12	no BA. only 0.5×10^5 BC	****	2.3±0.4
13	positive control (only BA)	33.0±0.2	31.3±4.3
14	negative control (H_2O)	-	-

* 16S-BA-allele concentration constant at 100 copies per reaction; ** averages (Ø) ± standard deviations (SD) of n = 3 tests; *** value at the end of the 42th PCR cycle; **** no regular amplification, no CT-value.

Table S10: Sensitivity panel - target organism *B. anthracis*.

# of *B. anthracis* strains	Phylogeny*	PCR result**
1	C.Br. A1055	positive
3	B.Br. CNEVA	positive
1	B.Br. Kruger B	positive
5	A.Br. 001/002	positive
1	A.Br. 011/009; A.Br.118 (STI)	positive
1	A.Br. 005/006	positive
1	A.Br. Vollum	positive
1	A.Br. Aust 94	positive
5	A.Br. Aust 94; A.Br.014	positive
1	A.Br. Aust 94; A.Br.015	positive
2	A.Br. 008/011; A.Br.127 (Pstr)	positive
1	A.Br. 008/011; A.Br.127(BUL)	positive
1	A.Br. 008/011; A.Br.161 (Heroin)	positive
1	A.Br. WNA	positive

* phylogeny according to (1) and (2); ** results of n = 3 tests.

Table S11: Specificity panel - potentially cross-reacting organisms.

Organism	Strain number	PCR result**
B. cereus	ATCC 10987	negative
B. cereus	2998	negative
B. cereus	3093	negative
B. cereus	LGL 3094	negative
B. cereus	ATCC 4342	negative
B. cereus bv. anthracis	CI-1	negative
B. cereus bv. anthracis	CA-1	negative
B. cereus	ATCC 33019	negative
B. thuringiensis	ATCC 10792	negative
B. thuringiensis	DSM 046	negative
B. paranthracis	2002	negative
B. weihenstephanensis	B-0293	negative
B. mycoides	B-298	negative
B. subtilis	ATCC 6091	negative
B. megaterium	ATCC 14581	negative
*Homo sapiens****	n.a.*	negative
*Bos taurus****	n.a.*	negative
*Capra aegagrus hircus****	n.a.*	negative
*Ovis gmelini aries****	n.a.*	negative
*Equus caballus****	n.a.*	negative

* not applicable (n.a.); ** results of n = 3 tests; ***typical host organisms.

Table S12: Specificity panel – organisms relevant for differential diagnostics and other pathogens.

Organism	Strain number	PCR result*	Organism	Strain number	PCR result*
Brucella sp.	F-070660	negative	*Moraxella catarrhalis*	B-0433	negative
Burkholderi mallei	L3-2962	negative	*Neisseria meningitidis*	B-1332	negative
Burkholderi pseudomallei	L3-0711	negative	*Propionibacterium acnes*	B-0438	negative
Burkholderia thailandensis	B-1668	negative	*Pseudomonas aeruginosa*	B-0040	negative
Campylobacter jejuni	B-1229	negative	*Serratia marcescens*	B-0014	negative
Candida albicans	B-1266	negative	*Sphingomonas zeae*	JM-791	negative
Citrobacter freundii	B-0022	negative	*Staphylococcus aureus*	B-0946	negative
Clostridium paraperfringens	B-1435	negative	*Staphylococcus epidermidis*	B-0026	negative
Clostridium sporogenes	B-1450	negative	*Stenotrophomonas maltophila*	B-0055	negative
Eikenella corrodens	B-0614	negative	*Streptococcus pneumoniae*	B-0847	negative
Escherichia coli	B-1324	negative	*Streptococcus pyogenes*	B-0846	negative
Francisella tularensis holarctica	F-0049	negative	*Vibrio cholerae*	B-1302	negative
Haemophilus influenzae	B-0850	negative	*Yersinia enterocolitica*	B-0099	negative
Klebsiella pneumoniae	B-0008	negative	*Yersinia pestis*	EV-76	negative
Legionella pneumophila	B-1341	negative	*Monkey Pox Virus*	MSF-6	negative
Listeria monocytogenes	DSM-12464	negative	*Vaccinia Virus*	VACV-0273/2004	negative
			Varicella Zoster Virus	none	negative

* results of n = 3 tests.

Table S13: Linearity of the 16S rRNA SNP-PCR*.

[Template]**	Assays day 1			Assays day 2			Assays day 3			(Ø±SD)***
**A.**										
10^9	9.3	9.4	9.5	9.7	9.8	9.8	9.1	9.1	9.1	9.4±0.3
10^8	12.6	12.6	12.5	13.0	13.1	13.0	12.3	12.3	12.3	12.6±0.3
10^7	16.0	16.0	16.0	16.7	16.6	16.6	15.8	15.8	15.9	16.1±0.3
10^6	19.2	19.1	19.0	19.5	19.6	19.6	18.8	18.8	18.8	19.2±0.3
10^5	22.6	22.7	22.6	23.0	23.1	23.2	22.5	22.3	22.3	22.7±0.3
10^4	25.9	25.8	26.0	26.2	26.3	26.3	25.6	25.6	25.6	25.9±0.3
10^3	29.5	29.4	29.6	30.0	29.9	29.9	29.2	29.1	29.0	29.5±0.3
10^2	32.3	32.9	32.3	32.9	33.0	32.8	32.4	32.2	32.0	32.5±0.3
10^1	35.8	35.7	36.2	36.1	35.8	35.8	35.9	36.2	35.8	35.9±0.2
10^0	36.9	37.3	38.8	39.2	38.9	38.6	-	37.2	-	38.1±1.0
**B.**										
10^7	17.1	17.0	17.1	16.7	16.8	16.8	16.8	16.8	16.8	16.9±0.2
10^6	20.4	20.7	20.4	20.2	20.2	20.2	20.3	20.3	20.3	20.3±0.2
10^5	24.0	24.0	24.1	23.8	23.7	23.7	23.8	23.7	23.9	23.9±0.2
10^3	27.4	27.3	27.5	26.9	27.0	26.8	27.0	27.1	27.1	27.1±0.2
10^2	30.8	29.6	30.7	30.5	30.5	30.6	30.5	30.7	30.4	30.5±0.3
10^1	34.0	34.1	33.8	33.7	33.7	33.8	33.6	34.0	33.9	33.8±0.2
10^0	38.3	36.7	37.1	36.1	36.9	36.7	37.3	37.1	36.6	37.0 ±0.6

* Replicates (n=3) of each PCR reaction with target DNA were run on three consecutive days; ** templates at indicated concentration per reaction were cloned fragment (A) or *B. anthracis* Ames DNA (B); *** averages (Ø) ± standard deviations (SD) of n = 3 x 3 tests.

Table S14: Probit-analysis of the 16S rRNA SNP-PCR.

Copies/reaction	# of tests	# of positive tests
10	12	12
8	12	12
6	12	12
4	12	12
3	12	12
2	12	7
1	12	4

Table S15: Linearity of the 16S rRNA SNP RT-PCR*.

[Template]**	Triplicates			(Ø±SD)***
10^8	10.2	9.8	10.4	10.1±0.3
10^7	13.7	13.7	13.8	13.7±0.1
10^6	16.7	16.7	16.8	16.7±0.1
10^5	20.0	19.5	21.2	20.2±0.9
10^4	24.5	24.3	23.8	24.2±0.3
10^3	26.6	26.9	26.6	26.7±0.2
10^2	29.8	31.2	30.9	30.6±0.7
10^1	34.7	35.0	34.9	34.8±0.2
10^0	40.3	37.0	40.0	39.1±1.8

* Replicates (n=3) of each PCR reaction with target DNA were run; ** templates at indicated concentration per reaction were RNAs (including genomic) DNA of *B. anthracis* Sterne; *** averages (Ø) ± standard deviations (SD) of n = 3 tests.

Table S16: Probit-analysis of the 16S rRNA SNP RT-PCR.

Copies/reaction	# of tests	# of positive tests
15	12	12
12	12	12
9	12	12
7.5	12	12
6	12	10
4.5	12	10
3	12	8
1.5	12	8
0	12	0

6.2. Supplementary Figures

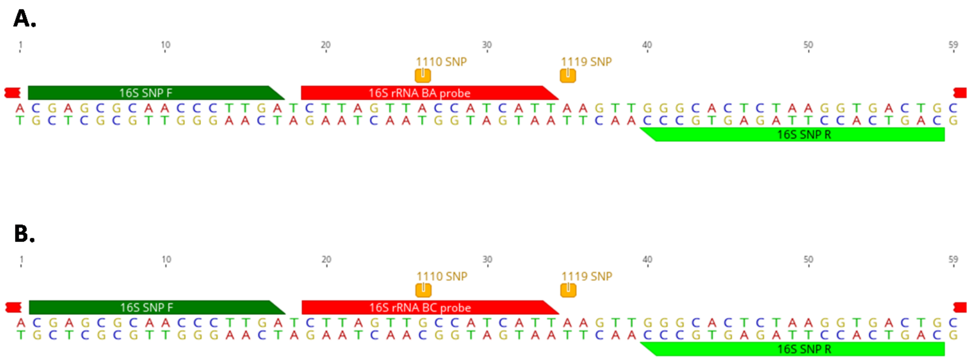

Figure S1: Partial sequence of different alleles of the 16S rRNA genes of *B. anthracis* with specific SNP (“A” at position 1110 in *B. anthracis* Ames Ancestor NC_007530; position 1139 according to (3))* and primer and probe positions. (A) *B. anthracis*-specific 16S-BA-allele. (B) alternative 16S rRNA gene allele (16S-BC-allele) in *B. anthracis* common for the *B. cereus*-group with alternative SNP state (“G” at position 1110 in *B. anthracis* strain Ames Ancestor, NC_007530). *a second SNP at position 1119 present in some of the 16S rRNA gene alleles was found not to be specific for *B. anthracis* (3).

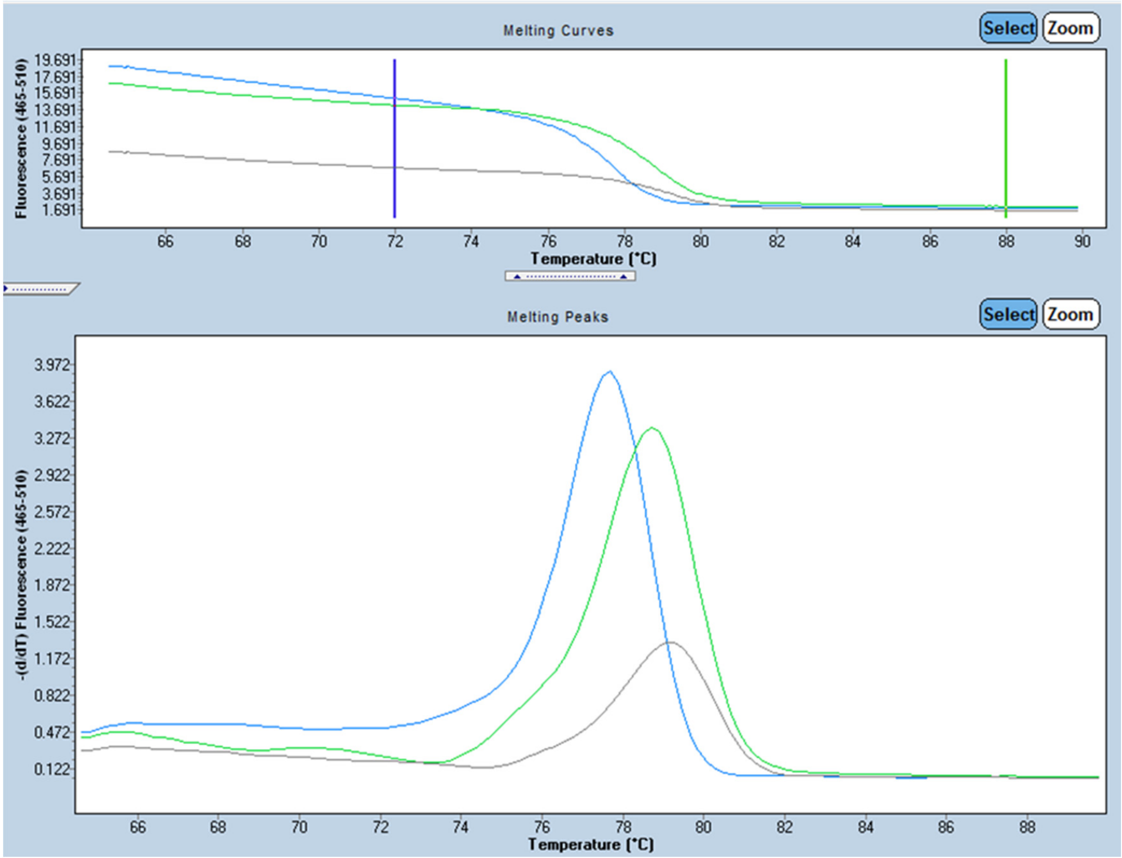

Figure S2: Melting point analysis of PCR products. Shown are melting point analyses as fluorescence change (upper panel) or as its first derivative (lower panel) over a temperature gradient. Amplificates were 16S-BA-allele (100 template copies, blue line), 16S-BC-allele (100 template copies, green line) or water-only control (grey line).

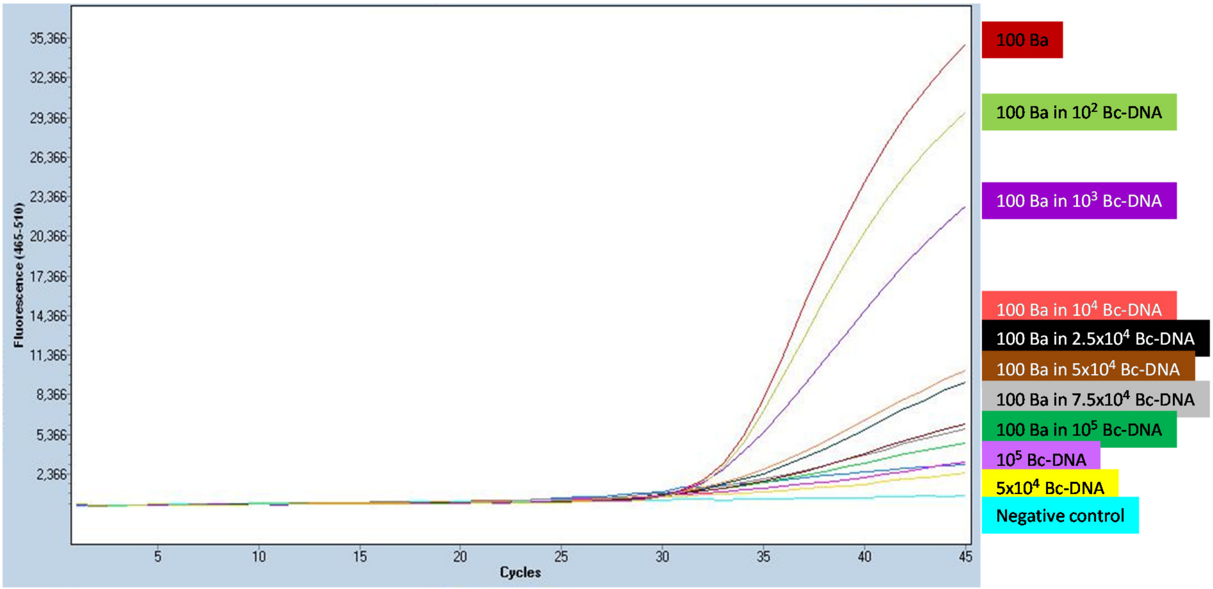

Figure S3: Competitive inhibition the 16S rRNA SNP-PCR assay by the alternative 16S-BC-allele. A constant 100 template copies of the 16S-BA-allele per reaction were titrated against increasing copy numbers of the alternative 16S–BC-allele and fluorescence recorded (shown are representative curves; see Table S9 for numerical data as replicates).

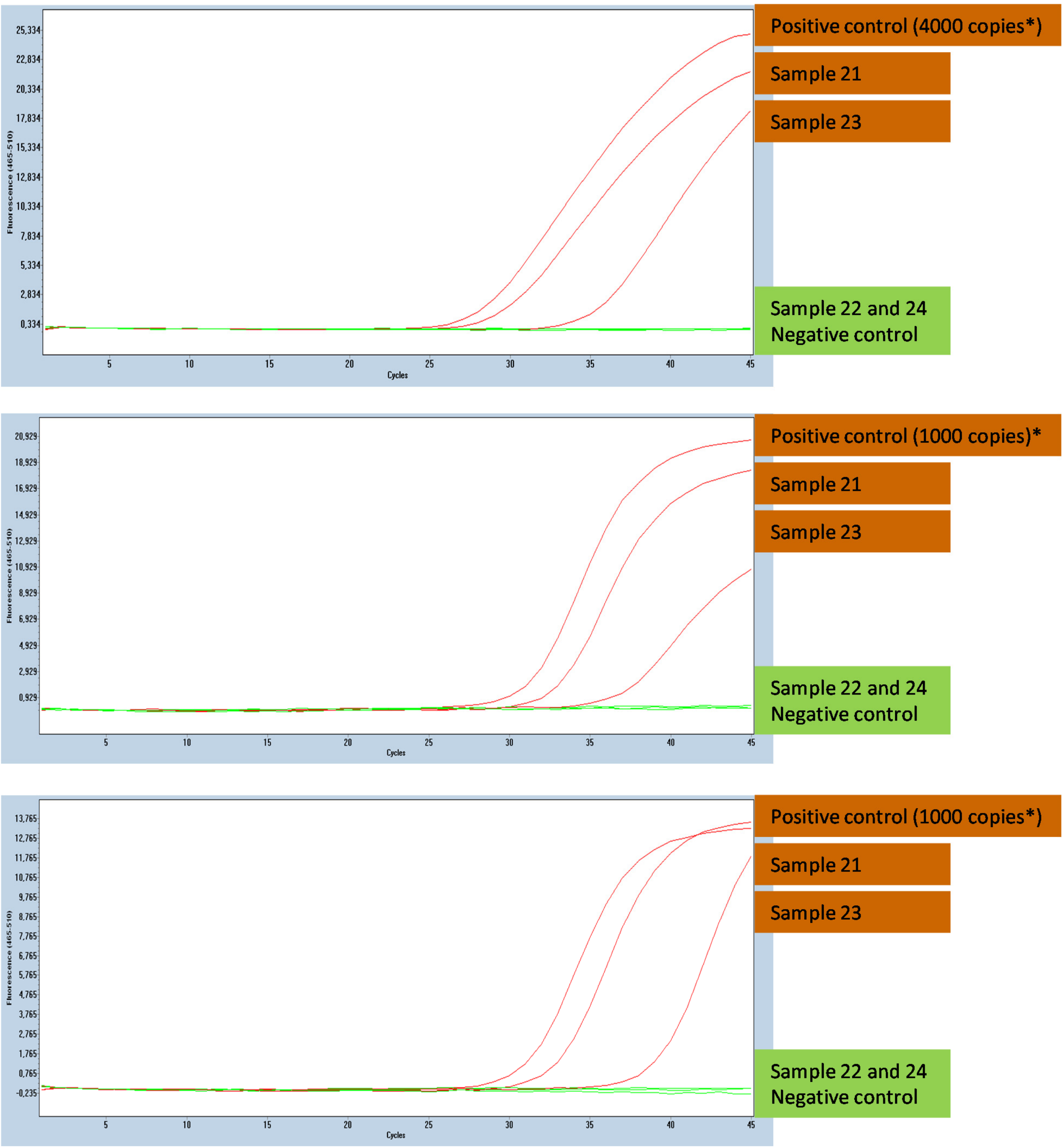

Figure S4: Challenge of the new 16S rRNA-based multi-copy assay with samples from a ring trial. Samples were subjected to real time PCR using the new 16S rRNA SNP assay (upper panel), published *dhp61* gene assay (4) (middle panel) or published *PL3* gene assay (5) (lower panel). Representative amplification curves (from n=3 with similar results) are shown. *Positive controls were 1000 genomes of *B. anthracis* Ames (harboring 4000 copies of the 16S-BA-allele but only 1000 copies of *dhp61* or *PL3*, respectively.

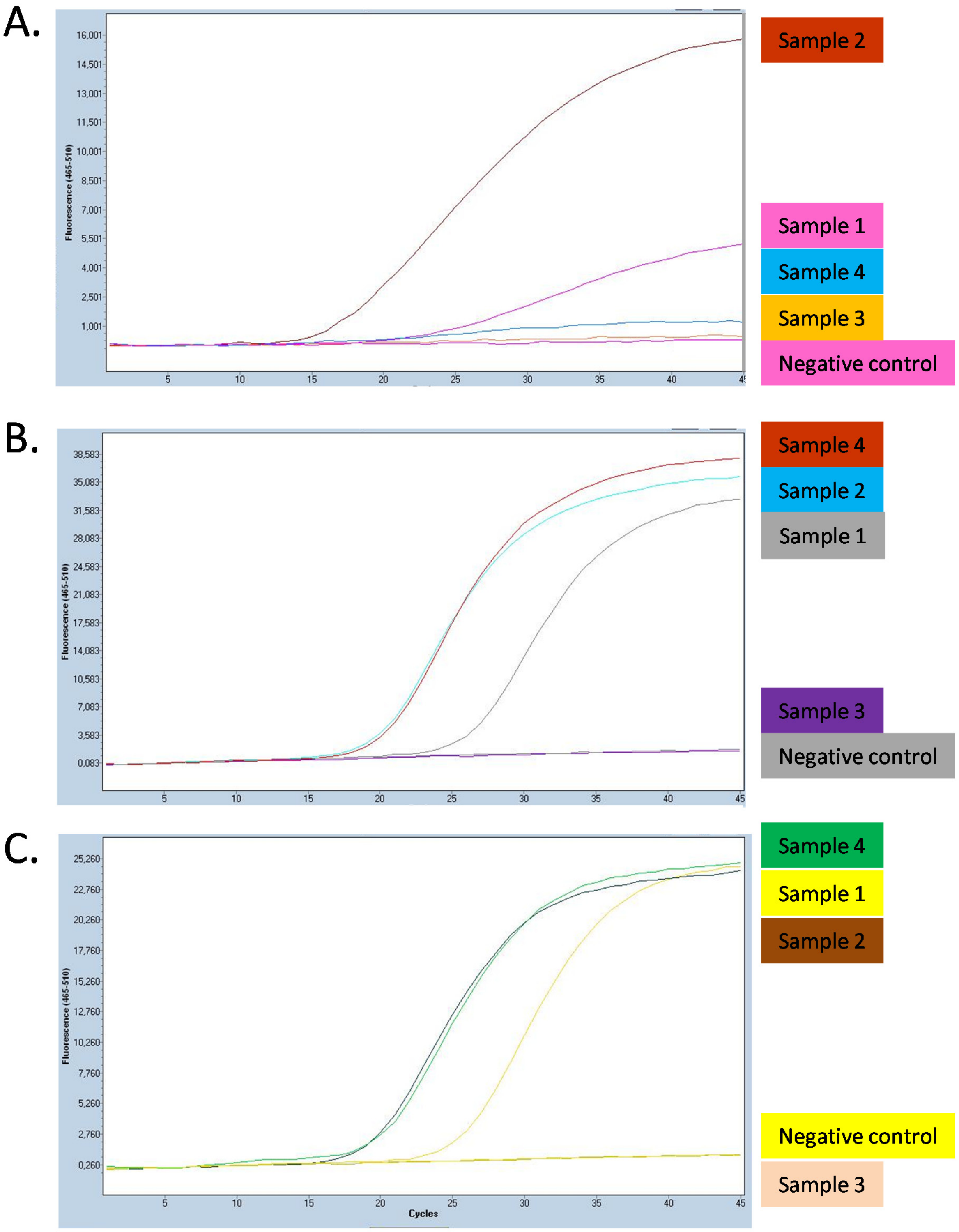

Figure S5: Challenge of the new 16S rRNA-based multi-copy assay with total DNA from spiked soil. Samples were subjected to real time PCR using the new 16S rRNA SNP assay (upper panel), published *dhp61* gene assay (4) (middle panel) or published *PL3* gene assay (5) (lower panel). Representative amplification curves (from n=3 with similar results) are shown.

References

1. Sahl JW, Pearson T, Okinaka R, Schupp JM, Gillece JD, Heaton H, Birdsell D, Hepp C, Fofanov V, Noseda R, Fasanella A, Hoffmaster A, Wagner DM, Keim P. 2016. A *Bacillus anthracis* genome sequence from the Sverdlovsk 1979 autopsy specimens. MBio 7:e01501-16.
2. Antwerpen M, Beyer W, Bassy O, Ortega-García MV, Cabria-Ramos JC, Grass G, Wölfel R. 2019. Phylogenetic placement of isolates within the Trans-Eurasian clade A.Br.008/009 of *Bacillus anthracis*. Microorganisms 7:689.
3. Hakovirta JR, Prezioso S, Hodge D, Pillai SP, Weigel LM. 2016. Identification and analysis of informative single nucleotide polymorphisms in 16S rRNA gene sequences of the *Bacillus cereus* group. J Clin Microbiol doi:JCM.01267-16 [pii] 10.1128/JCM.01267-16.
4. Antwerpen MH, Zimmermann P, Bewley K, Frangoulidis D, Meyer H. 2008. Real-time PCR system targeting a chromosomal marker specific for *Bacillus anthracis*. Mol Cell Probes 22:313-5.
5. Wielinga PR, Hamidjaja RA, Agren J, Knutsson R, Segerman B, Fricker M, Ehling-Schulz M, de Groot A, Burton J, Brooks T, Janse I, van Rotterdam B. 2011. A multiplex real-time PCR for identifying and differentiating *B. anthracis* virulent types. Int J Food Microbiol 145 Suppl 1:S137-44.
